# Prolactin Secretion in Healthy Adults Is Determined by Gender, Age and Body Mass Index

**DOI:** 10.1371/journal.pone.0031305

**Published:** 2012-02-17

**Authors:** Ferdinand Roelfsema, Hanno Pijl, Daniel M. Keenan, Johannes D. Veldhuis

**Affiliations:** 1 Department of Endocrinology and Metabolic Diseases, Leiden University Medical Center, Leiden, The Netherlands; 2 Department of Statistics, University of Virginia, Charlottesville, Virginia, United States of America; 3 Endocrine Research Unit, Mayo Medical and Graduate Schools, Clinical Translational Research Center, Mayo Clinic, Rochester, Minnesota, United States of America; Postgraduate Medical Institute & Hull York Medical School, University of Hull, United Kingdom

## Abstract

**Background:**

Prolactin (PRL) secretion is quantifiable as mean, peak and nadir PRL concentrations, degree of irregularity (ApEn, approximate entropy) and spikiness (brief staccato-like fluctuations).

**Hypothesis:**

Distinct PRL dynamics reflect relatively distinct (combinations of) subject variables, such as gender, age, and BMI.

**Location:**

Clinical Research Unit.

**Subjects:**

Seventy-four healthy adults aged 22–77 yr (41 women and 33 men), with BMI 18.3–39.4 kg/m^2^.

**Measures:**

Immunofluorometric PRL assay of 10-min samples collected for 24 hours.

**Results:**

Mean 24-h PRL concentration correlated jointly with gender (P<0.0001) and BMI (P = 0.01), but not with age (overall R^2^ = 0.308, P<0.0001). Nadir PRL concentration correlated with gender only (P = 0.017) and peak PRL with gender (P<0.001) and negatively with age (P<0.003), overall R^2^ = 0.325, P<0.0001. Forward-selection multivariate regression of PRL deconvolution results demonstrated that basal (nonpulsatile) PRL secretion tended to be associated with BMI (R^2^ = 0.058, P = 0.03), pulsatile secretion with gender (R^2^ = 0.152, P = 0.003), and total secretion with gender and BMI (R^2^ = 0.204, P<0.0001). Pulse mass was associated with gender (P = 0.001) and with a negative tendency to age (P = 0.038). In male subjects older than 50 yr (but not in women) approximate entropy was increased (0.942±0.301 *vs.* 1.258±0.267, P = 0.007) compared with younger men, as well as spikiness (0.363±0.122 *vs.* 0463±2.12, P = 0.031). Cosinor analysis disclosed higher mesor and amplitude in females than in men, but the acrophase was gender-independent. The acrophase was determined by age and BMI (R^2^ = 0.186, P = 0.001).

**Conclusion:**

In healthy adults, selective combinations of gender, age, and BMI specify distinct PRL dynamics, thus requiring balanced representation of these variables in comparative PRL studies.

## Introduction

Prolactin (PRL) is a 23 kDa protein secreted by the pituitary gland and has many actions of which the lactotrophic function is best known. This hormone has various other effects on reproduction, metabolism and tumorigenicity mainly described in animals, but the precise role in the human is less well established [1], [2].

Prolactin secretion proceeds via combined pulsatile (burst-like) and basal (time-invariant) modes of release. A complicating issue in defining normative ranges even for measures of PRL secretion is that they may depend upon one or more biological or clinical factors, such as gender, age, BMI, sex-steroid concentrations, core temperature, nutrition, stress, exercise, medications and renal disease [1], [3]–[9]. Prolactin secretion is primarily regulated by the inhibitory action of hypothalamic dopamine, and by ultrashort autofeedback, but the physiological role of various releasing hormones **is** not established in man [2]. These factors would putatively determine more complex PRL dynamics, which arise physiologically from feedforward (stimulatory) and feedback (inhibitory) signals interacting in an integrative fashion, as demonstrated in detail for GH and LH in men [10]. Novel integrative measures are approximate entropy (ApEn) and spikiness, which reflect the complexity and stability of signaling interactions in homeostatic systems [10].

Other pituitary hormone systems, including GH, TSH, ACTH and LH exhibit age-, gender-, and BMI-related changes [11]–[13]. Although PRL levels are generally lower in men than in women, the influence of aging and adiposity is less well investigated, especially in men. The small size of most cohorts evaluated to date, the narrow age and BMI ranges encompassed, and the lack of inclusion of both genders collectively make the possibility of statistical type I or type II errors high in earlier studies. As importantly, because of correlations among age, BMI and gender, multivariate regression is needed for definitive inferences. Nonetheless, multivariate analysis also is unreliable in small cohorts. To overcome these obstacles would require investigation of a large number of healthy adults, both men and women, over wide ranges of age and BMI. In this light, the present study examines the dependencies of PRL release (mean, peak, nadir, 24-h secretion, ApEn, spikiness) on individual and/or combined clinical characteristics in 74 healthy individuals sampled frequently (every 10 min) for a sufficiently representative duration (24 h) and analyzed with a high-sensitivity PRL assay (immunofluorometric platform).

## Methods

### Clinical protocol

The cohort of healthy individuals studied in this project, originated from different studies, in which they served as controls, including studies on PRL secretion in patients with prolactinoma, obese subjects and patients with neurological disorders [3], [14]–[18]. In these studies the women and men volunteered for and completed the sampling study. Subjects originated from the same community, and were evaluated in an identical sampling paradigm and PRL assay (below). Informed written consent was obtained from the subjects in all these published studies and the studies were approved, including this retrospective analysis, by the ethics committee of the Leiden University Medical Centre. All analyses reported here used techniques not previously applied in any of the published studies. Clinical characteristics of the volunteers (41 women and 33 men) are listed in Table 1. Postmenopausal individuals studied here did not use estrogen therapy. Premenopausal women were studied in the follicular phase of the menstrual cycle.

**Table 1:** Baseline subject characteristics.

	Women (41)	Men (33)	P-value
Age (yr)	40 (22–77)	42 (21–77)	0.97
BMI (kg/m^2^)	27.5 (18–39)	25.2 (21–36)	0.01
IGF-I (nmol/liter)	18 (10–35)	18.1 (9.9–32.1)	0.80
Estradiol (pmol/liter}	98 (5–297)	46 (24–90)	<0.0001
Testosterone (nmol/liter)	0.60 (0.10–1.2)	16 (12.5–23.1)	<0.0001
Free T_4_ (nmol/liter)	14.7 (12–18.6)	16 (12.5–23.1)	0.04
Mean PRL (µg/liter)	6.2 (2.0–18.4)	4.1 (2.1–9.7)	<0.0001
Peak PRL (µg/liter)	17.4 (7.1–38.2)	9.5 (4.9–25.1)	<0.0001
Nadir PRL (µg/liter)	2.8 (0.6–9.4)	1.9 (0.75–5.7)	0.005
PRL ApEn (unitless)	0.895 (0.270–1.807)	1.038 (0.400–1.913)	0.42
Spikiness (unitless)	0.326 (0.207–0.911)	0.361 (0.208–0.778)	0.24

Data are shown as median and range. Statistical evaluation was done with the Kolmogorov-Smirnov test.

Participants maintained conventional work and sleeping patterns and reported no recent (within 10 days) transmeridian travel, weight change (>2 kg in 6 weeks), shift work, psychosocial stress, prescription medication use, substance abuse, neuropsychiatric illness, or acute or chronic systemic disease. A complete medical history, physical examination, and screening biochemistry tests were normal. Volunteers were admitted to the Study Unit the evening before sampling for adaptation. Ambulation was permitted to the lavatory only. Vigorous exercise, daytime sleep, snacks, caffeinated beverages, and cigarette smoking were disallowed. Meals were provided at 0900, 1230 and 1730 h, and room lights were turned off between 2200 and 2400 h, depending upon individual sleeping habits. Blood samples (2.0 mL) were withdrawn at 10-min intervals for 24 h. Total blood loss was less than 360 mL. Volunteers were compensated for the time spent in the study.

### Assays

Plasma PRL concentrations were measured with a sensitive time-resolved fluoroimmunoassay (Wallac Oy, Turku, Finland). The limit of detection (defined as the value 2 SD above the mean value of the zero standard) was 0.04 µg/L. The assay was calibrated against the 3rd WHO standard 84/500. The intra-assay coefficient of variation (CV) varied from 3.0–5.2% in the assay range 0.1–250 µg/L, with corresponding interassay CV's of 3.4–6.2%. Serum estradiol concentrations were assayed by a sensitive RIA (Spectria Estradiol Sensitive RIA, Orion Diagnostica, Espoo, Finland).The detection limit of the assay is 5 pmol/L. The intraassay CV was 21% at concentrations below 30 pmol, 4.5% at 85 nmol/L and 1.7% at 200 pmol/L. Testosterone was measured by RIA (Siemens Healthcare Diagnostics, Deerfield, Il, USA). The detection limit is 0.2 nmol/L. The intraassay CV at 1 nmol/L is 20% and at 14 nmol/L 12%. Free thyroxine was measured by electrochemoluminescence immunoassay (Elecsys 2010, Roche Diagnostics, Almere, The Netherlands). The detection limit is 0.6 pmol/L and the intraassay CV range amounts 5–8%. Serum IGF-I concentration was measured with the Immulite 2500 system (Diagnostic Products Corporation, Los Angeles, CA, USA). The detection limit is 1.5 nmol/L. The intra-assay variation was 5.0 and 7.5% at levels of 8 and 75 nmol/L, respectively. Simple mean, peak and nadir PRL concentrations Mean (24-h average), peak (single daily maximum) and nadir (single daily minimum) PRL concentrations were determined in each subject.

### Deconvolution analysis

**Figure 1:**
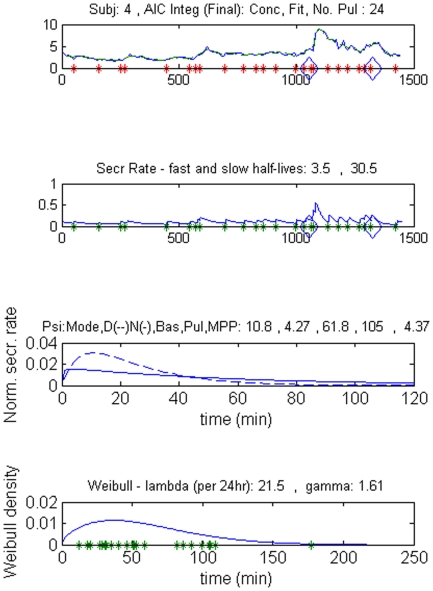
Part of the graphic output of the deconvolution analysis in a male control subject, the upper panel shows the original serum PRL concentrations (µg/L) and the fitted concentration curve (interrupted line). Asterisks denote pulse onsets, and the rhomboids the time of waveform switch. Time 0 min is 0900 hr. The second panel shows the secretion rate in µg/L.min. The third panel represents the secretion rate within bursts (normalized secretion over time) for the daytime (interrupted line) and nighttime (continuous line), stressing the difference in time at which the maximal secretion rate is reached. The lowest plot shows the statistical distribution of the interpulse delays.

Prolactin concentration time series were analyzed via a recently developed automated deconvolution method, empirically validated using hypothalamo-pituitary sampling and simulated pulsatile time series [19]–[22]. The Matlab-based algorithm first detrends the data and normalizes concentrations to the unit interval [0, 1]. Second, the program creates multiple successive potential pulse-time sets, each containing one burst less via a smoothing process (a nonlinear adaptation of the heat-diffusion equation). Third, a maximum-likelihood expectation estimation method computes all secretion and elimination parameters simultaneously conditional on each of the multiple candidate pulse-time sets. Deconvolution parameters comprise basal secretion (β_0_), two half-lives (*α*_1_,*α*_2_), secretory-burst mass (*η*_0_, *η*_1_), random effects on burst mass (*σ*_A_), measurement error (*σ_ε_*), and a three-parameter flexible Gamma-secretory-burst waveform (β_1_, β_2_, β_3_). The unit-area normalized shape of secretory bursts (plot of rate of secretion over time) was permitted to differ in the day and night, thus constituting a dual-waveform model of secretion. Two change point times were estimated to demarcate onset of the day and onset of the nighttime waveforms within each 24-h pulse train. For PRL, the fast half-life was represented as 3.5 min constituting 37% of the decay amplitude. The slow half-life was estimated as an unknown variable between 20 and 50 min [23], [24]. All candidate pulse-time sets were deconvolved. Statistical model selection was then performed to distinguish among the independently framed fits of the multiple candidate pulse-time sets using the Akaike information criterion. Observed interpulse intervals were described by a two-parameter Weibull process (more general form of a Poisson process, which uncouples the mean from the variance).The parameters (and units) are frequency (number of bursts per total sampling period, lambda of Weibull distribution), regularity of interpulse intervals (unitless gamma of Weibull), slow half-life (minutes), basal and pulsatile secretion rates (concentration units/session), mass secreted per burst (concentration units), and waveform shape (mode, or time delay to maximal secretion after objectively estimated burst onset, in min). A typical example of part of the graphic output of the deconvolution calculations is shown in Fig. 1.

### ApEn

Approximate Entropy (ApEn) is a sensitive and specific statistic for discriminating insidious differences in serial dynamics. ApEn is calculated for any time series as a single nonnegative number, with zero denoting perfect orderliness, as for a sine wave, and larger ApEn values corresponding to more apparently irregular dynamics [25]. The ApEn metric evaluates the consistency of recurrent subordinate (non-pulsatile) patterns in the data, and thus yields information distinct from and complementary to deconvolution (pulse) analyses [10], [25]. In typical biological applications, ApEn calculations are normalized against the SD of the data series by defining a pattern-reproducibility threshold value of *r* = 0.2 SD validated for data lengths *n*≥60 samples [26]. This choice of *r* limits random effects of low measurement variability (typically ≤0.065 SD), thus allowing discrimination between fine gradations in the orderliness of the underlying process. Validation studies have established the suitability of the *m* = 1 as the pattern-recurrence length for time series comprising 60<*n*<300 points, as would be true for many endocrine profiles [26]. For this (*m*, *r*) pair, there is quantifiably greater regularity (lower ApEn) of nocturnal GH secretion in adult male than female rats castrated prepubertally, as well as in normal men and women [27]. ApEn is translation- and scale-independent mathematically, which means that adding or multiplying each data value by a fixed number does not alter ApEn [28]. This feature ensures valid comparisons between different mean concentrations, overall variation, or secretion rates due to age, gender, physiological state, and pathology. For example, more irregular (higher ApEn, less orderly) GH secretion occurs in patients with either hypersomatotropism due to GH-secreting pituitary tumors or hyposomatotropism due to hypopituitarism despite 1000-fold differences in GH production [29], [30].

### Spikiness

Spikiness was defined as the ratio of the SD of the first-differenced (incremental) time series to the SD of the original series [31]. Spikiness quantifies the extent of sharp, brief, staccato-like unpatterned fluctuations.

### Cosinor analysis

The diurnal variation of PRL was analyzed by a non-linear cosine approximation. Measures are the mesor (average level around which the 24-hour oscillation occurs), amplitude (half of the difference between the highest and lowest values) and acrophase (time of the maximum).

### Statistical analysis

Stepwise forward-selection multivariate linear regression analysis of untransformed PRL measures was used to examine correlations between preselected PRL parameters (individual dependent variables) and one or more of age, BMI, gender and serum hormone concentrations (independent variables). Statistical comparisons by gender were carried out via the nonparametric Kolmogorov-Smirnov test. Significant contrasts were confirmed by unpaired two-tailed Student's t-test of log-transformed PRL-deconvolution measures. In addition, ANOVA was used for comparisons of more than 2 groups. Data are given as median and absolute range, and as mean and standard deviation or standard error. Analyses used Systat, version 11 (SPSS Inc., Chicago, IL, USA). *P*<0.05 was considered significant.

## Results

Table 1 shows the median (absolute range) subject characteristics for the 33 men and 41 women. Their ages were similar, but the BMI was higher in women than men. Estradiol and testosterone concentrations were as expected for the genders, but free thyroxine was higher in men than women and within normal limits, while IGF-I concentrations did not differ between genders. Mean 24-h, peak and nadir PRL concentrations were all larger in women than in men. ApEn (regularity) and spikiness (brief sharp elevations) were similar in men and women (Table 1).

**Figure 2:**
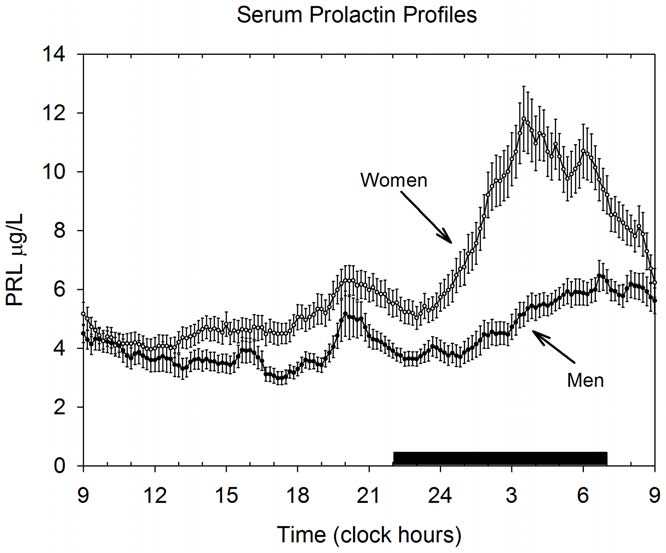
Twenty-four hour serum PRL concentration profiles in 41 healthy women and 33 healthy men. Blood samples were drawn every 10 min. Data are shown as mean, and the bars represent the SEM. The period with lights off (shown as the black horizontal bar) was between 2300 and 0700 h.

The serum PRL profiles across the 24 hour cycle are displayed in Fig. 2, showing the marked gender differences, especially during the phase with lights off.

**Figure 3:**
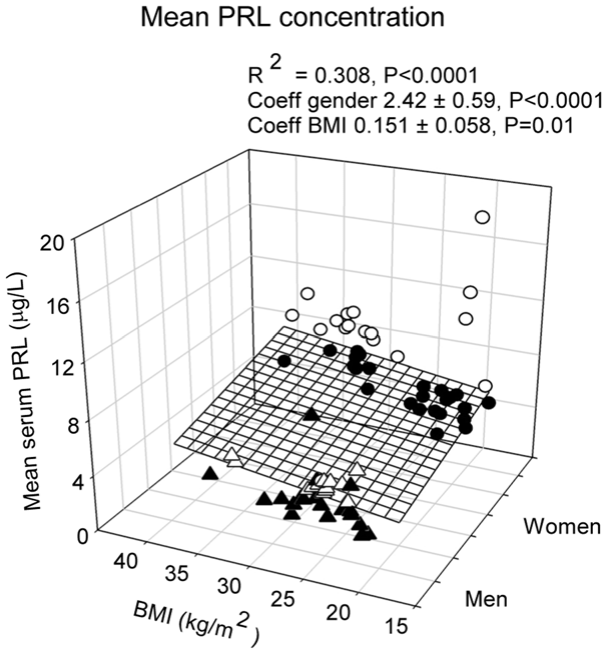
Multiple linear regression between age, gender and mean serum PRL concentration. Data were obtained in 74 healthy subjects, who underwent 24-h blood sampling at 10-min intervals. Male subjects are shown as triangles, female subjects as circles. Data points above the regression plane are open, below they are closed.

**Figure 4:**
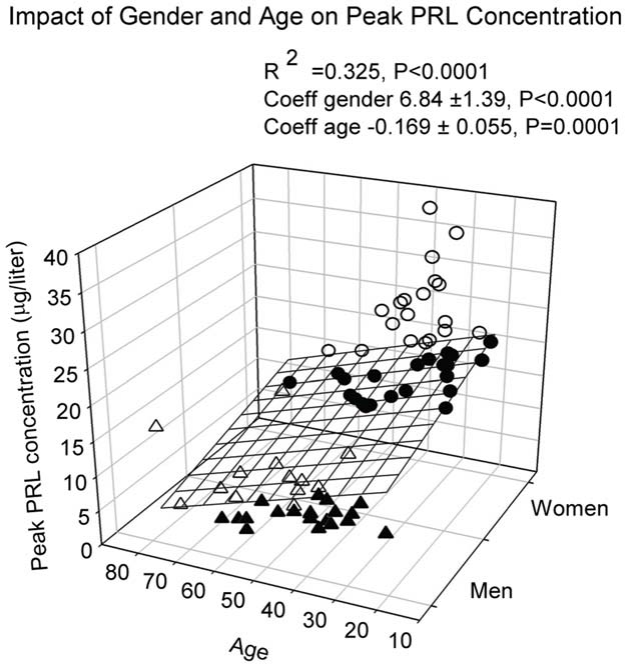
Multiple linear regression between age, gender and maximal PRL concentration in the 24-h serum profile. Data were obtained in 74 healthy subjects, who underwent 24-h blood sampling at 10-min intervals. Male subjects are shown as triangles, female subjects as circles. Data points above the regression plane are open, below they are closed.

Stepwise forward-selection multivariate regression analysis was employed to assess the association of individual PRL measures with gender, age and BMI. Mean 24-h PRL concentration was associated with gender (female>male, P<0.0001) and BMI (P = 0.01), but not with age (R^2^ = 0.308, ANOVA P<0.0001) (see Fig. 3). Nadir PRL concentration correlated with gender only (R^2^ = 0.077, P = 0.017). However, peak PRL concentration correlated with gender (P<0.0001) and negatively with age (P<0.0001), overall R^2^ = 0.325, P<0.0001 (see Fig. 4). PRL ApEn, a measure of secretory regularity, and spikiness, a metric of brief, staccato-like increases in secretion, were gender-, age- and BMI-invariant.

**Figure 5:**
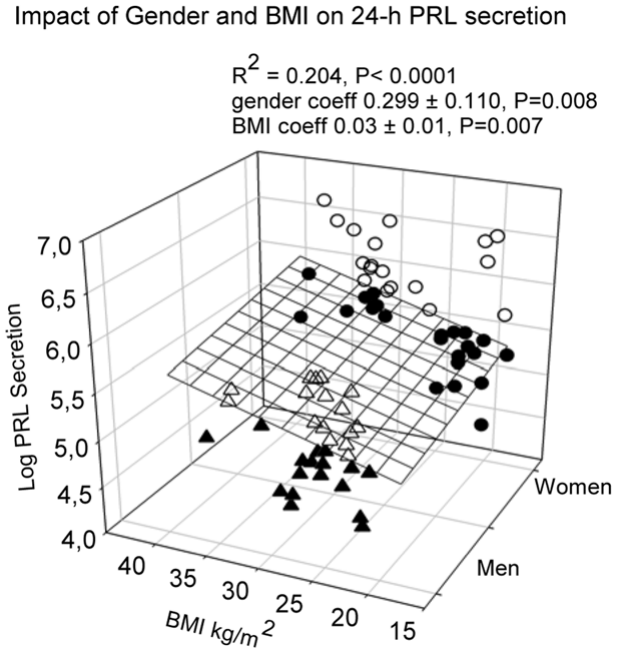
Multiple linear regression between age, gender and total logarithmically transformed PRL secretion, calculated by deconvolution analysis. Data were obtained in 74 healthy subjects, who underwent 24-h blood sampling at 10-min intervals. Male subjects are shown as triangles, female subjects as circles. Data points above the regression plane are open, below they are closed.

**Figure 6:**
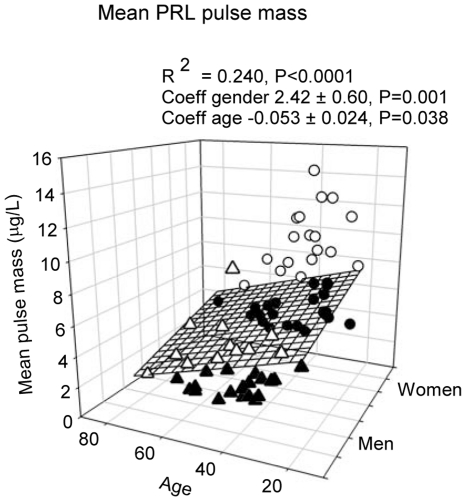
Multiple linear regression between age, gender and mean PRL pulse mass, calculated by deconvolution analysis. Data were obtained in 74 healthy subjects, who underwent 24-h blood sampling at 10-min intervals. Male subjects are shown as triangles, female subjects as circles. Data points above the regression plane are open, below they are closed.

Based on deconvolution analysis and on unpaired statistical comparisons, gender determined pulsatile PRL secretion (P<0.0001), total secretion (P<0.0001), but not basal secretion. Pulsatile secretion was amplified by **1.5** fold due to increased burst mass in women, with unchanged pulse frequency (Table 2). Forward-selection multivariate regression of PRL deconvolution results demonstrated that basal (nonpulsatile) secretion tended to be associated with BMI (R^2^ = 0.058, P = 0.03), pulsatile secretion with gender (R^2^ = 0.152, P = 0.003), and total secretion with gender and BMI (R^2^ = 0.204, P<0.0001, Fig. 5). Pulse mass was associated with gender (P = 0.001) and with a negative tendency to age (P = 0.038) (Fig. 6).

**Table 2:** Prolactin deconvolution parameters in men and women.

	All (n = 74)	Women (n = 44)	Men (n = 33)	K-S test	Student's t-test
Number of pulses (24 h^−1^)	19 (12–29)	19 (12–28)	19 (13–29)	0.80	0.82
Slow half-life (min)	34.6 (20–45)	34 (20–45)	34.8 (20–45)	0.52	0.85
Day mode (min)	9.8 (3–23.9)	9.2(3–21)	9.8 (3–23.9)	0.72	0.70
Night mode (min)	11.5 (3–30)	13.6 (3.1–30)	11.2 (3–17.4)	0.12	0.29
Basal secretion (µg/liter.24 h)	104 (91–530)	119 (9–530)	84(22–260)	0.15	0.41
Pulsatile secretion (µg/liter.24 h)	114(30–675)	138(60–675)	91(30–260)	0.002	<0.0001
Total secretion (µg/liter.24 h)	235(83–780)	284(83–780)	187(90–380)	0.009	<0.0001
Mass per burst (µg/liter)	6.1 (1.7–29.5)	7.4 (2.8–29.5)	4.6 (1.7–13.6)	0.01	<0.0001
Lambda(frequency/24 h,unitless)	17.8 (11.8–26.1)	17.8 (11.8–26.1)	17.8 (11.9–25.5)	0.96	0.92
Gamma (regularity, unitless))	1.92 (1.28–3.71)	1.95 (1.28–3.71)	1.88 (1.41–2.80)	0.51	0.30

Data are shown as median and range. Statistical comparisons were done with the Kolmogorov-Smirnov test and the unpaired Student's t-test after logarithmic transformation of the data.

We also compared results in premenopausal women with postmenopausal women. In postmenopausal women the following parameters were smaller than in women with a regular menstrual cycle: mean 24 h PRL, peak PRL, fasting PRL, nadir PRL, pulsatile secretion, mean pulse mass and total secretion (see Table 3). However, these parameters were not different in postmenopausal women and men (P-values lying between 0.27 and 0.75) (Table 3). In male subjects older than 50 yr, ApEn (0.942±0.301 *vs.* 1.258±0.267, P = 0.007) and spikiness (0.363±0.122 *vs.* 0.463±0.212, P = 0.031) were increased compared with values in younger men. Other parameters were statistically not different.

**Table 3:** Prolactin secretion characteristics in premenopausal and postmenopausal women, and men.

	Men	Pre MP women	Post MP women	ANOVA P-value	Men vs Post MP women	Pre MP vs Post MP
BMI	25.2±3.50	28.5±6.39	21.8±3.1	0.12		
Pulse frequency (nr/24 h)	19.5±4.0	18.9±4.5	32.4±9.1	0.13		
Slow half-life (min)	33.2±7.8	34.3±8.3	8.1±5.6	0.80		
Mode day (min)	10.9±5.2	10.4±5.1	10.4±5.4	0.29		
Mode night (min)	11.2±4.8	12.8±6.4	128±82	0.92		
Basal secretion (µg/L.24 h)	106±57	163±138	113±34	0.73		
Pulsatile secretion (µg/L.24 h)	101±46	203±147	241±82	<0.001	0.30	<0.001
Total secretion (µg/L.24 h)	207±82	366±188	5.17±1.77	<0.001	0.31	0.02
Mean pulse mass (µg/L)	5.3±2.53	10.6±1.17	19.5±3.5	<0.001	0.74	0.001
Lambda ( pulse frequency)	18.1±3.6	17.6±3.9	2.138±0.427	0.25		
Gamma (regularity, unitless)	1.943±0.352	2.017±0.433	1.023±0.337	0.06		
ApEn (unitless)	1.032±0.314	0.930±0.349	0.381±0.127	0.32		
Spikiness (unitless)	0.391±0.130	0.363±0.143	5.10±2.09	0.56		
Mean 24 h PRL (µg/L)	4.35±1.46	7.72±3.16	2.52±1.09	<0.001	0.27	0.001
Minimum PRL(µg/L)	2.13±1.03	3.49±2.16	11.3±4.56	0.005	0.46	0.08
Maximum PRL (µg/L)	10.7±4.04	20.3±7.07	3.88±1.42	<0.001	0.75	<0.001
Fasting PRL (µg/L)	4.18±1.51	7.01±5.17	3.88±1.42	0.001	0.48	0.002

Data are shown as mean and standard deviation.Comparisons between groups were made with ANOVA after logarithmic transformation of the data. Contrasts between groups were made only if the overall ANOVA was significant. MP: menopausal.

In the linear regression analyses of the PRL secretion parameters with age, BMI, gender and serum estradiol concentration, the last was an independent predictor of total PRL secretion (P = 0.002), basal secretion (P = 0.01), mean 24-h concentration (P = 0.002), minimal concentration (P = 0.002), but not of pulsatile secretion (P = 0.51), and maximal concentration (P = 0.32). Since we had testosterone values only in men, and with a few exceptions not in women, the regression analysis of the influence of testosterone was restricted to men. None of the parameters was related to the serum testosterone concentration.

**Figure 7:**
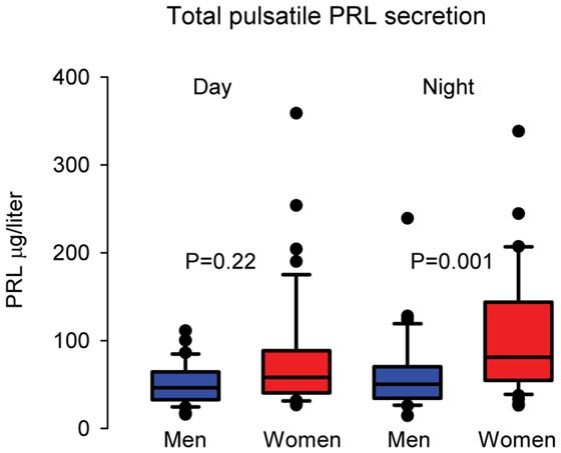
Box plots of the pulsatile PRL secretion during day time and during the period with lights off (2300 -0700 h). Differences between men and women were calculated with the Kolmogorov-Smirnov test.

Pulsatile PRL secretion was dependent on the diurnal cycle. During day time PRL secretion in men amounted 51.0 (14–239) µg/liter.24 h and in women 79.0 (26–358) µg/liter.24 h, P = 0.22. During the period with lights off these values were 61.0 (16–111) µg/liter.24 h in men and 111.0 (26–421) µg/liter.24 h in women, P = 0.001 (Fig. 7).

**Figure 8:**
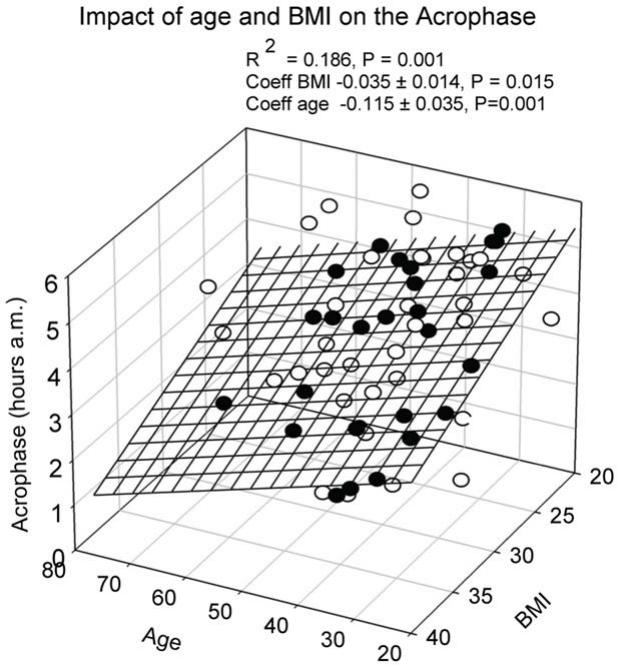
Multiple linear regression between age, BMI and the acrophase of the PRL rhythm. Data points above the regression plane are open, below the plane they are closed. There were no gender differences in acrophase.

All subjects had a significant diurnal PRL rhythm. The detailed results are displayed in Table 4. The mesor and amplitude were larger in women than men, but the time at which the maximal value was obtained did not differ between genders. The acrophase correlated negatively with BMI and age (R^2^ = 0.186, P = 0.003), see Fig. 8.

**Table 4:** Cosinor analysis of the 24-h serum prolactin profiles in healthy subjects.

	men	women	P-value
Mesor (µg/L)	3.90±0.25	5.91±0.47	<0.001
Amplitude(µg/L)	1.12±0.10	1.92±0.17	<0.001
Acrophase (clock hours a.m.	3.97±0.28	3.72±0.25	0.52

Data are shown as mean ± SEM. Statistical comparisons were made with the Student's t-test for unpaired data.

## Discussion

This study evaluated simple and complex measures of prolactin secretion in relation to gender (women ***vs*** men), age and BMI. Simple measures of prolactin secretion, *e.g.* nadir, peak and mean levels, as used in clinical settings, showed a strong relation to gender, age (decreased peak value only) and BMI (increased mean and nadir). Deconvolution analysis showed that pulsatile, but not basal (non-pulsatile), secretion was larger in women than men and caused by amplified mean burst mass with unchanged pulse frequency. Basal secretion was positively dependent on BMI and serum estradiol concentration.

Recent publications on 24-h secretion profiles of pituitary hormones in the human, with a 10-min sampling scheme, measured with robust and sensitive assays, and analyzed with operator-independent tools have demonstrated different impacts of gender, age and BMI in multivariate regression analysis. For instance, GH secretion in 100 adult subjects was determined independently by age, BMI and gender, thus decreasing by advancing age and increasing BMI, and with a higher secretion rate in women than men [11]. In contrast, ACTH secretion is larger in men than women, and positively correlated with BMI [13]. On the other hand, TSH secretion is gender invariant and only age-dependent in women [12].

The present study shows that PRL secretion is especially determined by gender and BMI. When analyzed separately postmenopausal women exhibited a 40% decrease in PRL secretion compared with premenopausal women in the follicular phase of their menstrual cycle. Healthy men did not show a decrease after age fifty, but rather exhibited a slight 18% increase, suggesting that age *per se* may not be the cause of the decrease in women. Therefore, studies on PRL physiology and pathophysiology should include a carefully balanced control group for meaningful comparisons.

One obvious cause for the decrease in PRL in women is the lack of estrogens in the menopausal phase, as suggested by the regression analysis in women. However the role of estrogens in regulating PRL secretion is certainly not straightforward. During the menstrual cycle no influence of different estradiol concentrations on PRL is demonstrable, but in this study only part of the 24 h cycle was analyzed [32]. Furthermore, the PRL increase after TRH injection is independent of the stage of the menstrual cycle [33]. However the value of such studies is limited because of the non-physiological TRH dose, which achieved PRL levels higher than those in spontaneous PRL secretory patterns. On the other hand, high dose orally administered estradiol in postmenopausal women, leading to greatly increased estradiol levels increases PRL. This does not occur after transdermal administration [34]. Other studies have even shown a decrease in PRL after transdermal estrogen administration in postmenopausal women [35], or no change [36]. However, it is conceivable that long-term effects of estrogens regulate the prolactin cell mass, thus explaining hypothetically the remarkable decrease in serum PRL concentration after menopause and possibly also the moderate increase in elderly men by enhanced bioavailable estradiol [37].

Interestingly, estradiol correlated with basal PRL secretion and the minimal 24-h concentration, and secondary to this the mean 24-h concentration and total secretion, but not with pulsatile secretion and the maximal value. This observation suggests a diminished restraint on secretion, but the mechanisms involved are currently not known. In addition, this observation also underlines a marked difference with GH secretion, where estrogens increase specifically pulsatile secretion [38], [39].The present result, however, should be confirmed by estradiol measurements with the more precise high performance liquid chromatography-tandem mass spectrometry [40] and free estradiol. Collectively, the impact of estrogens on PRL secretion is still not completely clear, partly because of nonphysiological experiments and partly because of correlation studies that do not prove a causal role. Potentially, blocking of the estrogen receptor by specific drugs may provide more insight into the physiological role of estrogens on PRL secretion.

Leptin is one of the various factors modulating PRL secretion and the administration restores lactation in the leptin-deficient ob/ob mice [41]. Infusion of leptin raises serum PRL concentration in fasted rats to levels present in normal fed animals [42]. Furthermore, a direct effect on PRL secretion by leptin on the pituitary *in vitro* has also been demonstrated [43]. Therefore, it is possible that age- and gender-dependent differences in serum leptin concentrations, *i.e* higher levels in women than men, and a 20% decrease after menopause, modulate PRL secretion as we describe in this study [44]. Unfortunately, leptin levels were measured only in a limited number of subjects, so that we could not investigate the relationship between this hormone and PRL secretion parameters.

Differences in PRL secretion between genders have rarely been investigated in relation to the diurnal cycle and not yet with deconvolution techniques. In this study the gender difference was only present during the sleep period but not during day time. This finding suggests that the dopamine restraint and putative stimulatory factors exhibit circadian properties, but also reveals the impact of gender.

Increased age is characterized by advance (earlier in day) shifting of acrophases of rhythms and diminished amplitude, possibly related to sleep fragmentation and earlier sleep onset and awakening [45]. In this study, age was weakly negatively correlated with PRL pulse mass and the maximal value attained during the 24-h rhythm. Studies which compared nocturnal PRL secretion in elderly and young subjects established decreased pulse amplitude in older age [46], [47]. On the other hand, PRL secretion during the daytime was age-invariant [46].The latter observation suggests that the decrease in nocturnal PRL secretion is not the result of decreased lactotrope cell mass. In another study in men aged 30–96 yr, non-fasting early afternoon serum PRL concentration increased slightly with advancing age, which was attributable to subjects older than 75 yr, whose data are not included in other investigations with a lesser age span [48]. Indeed, another analysis comparing basal and TRH-stimulated PRL concentrations in two age groups (23–45 *vs.* 56–75 yr) found comparable basal levels, but a negative correlation between age and stimulated PRL concentration [49]. Collectively, these observations are consistent with altered (increased) dopaminergic tonus in the elderly during the sleep period, and which can be restored by metoclopramide administration and diminished responsiveness of the prolactin-secreting cell to TRH [5]. Body composition is an important modulator of hormone secretion, as found for instance for insulin, leptin, TSH, ACTH and cortisol secretion. In this study, total PRL secretion was correlated with BMI in both genders. This result corroborates the notion of enhanced PRL secretion in obesity in most studies [3], [50], but not all [51]. Weight reduction by very low calorie diet or bariatric surgery diminished PRL secretion [52]–[54]. However, in a study by Ernst and colleagues no change in basal PRL levels was observed after massive weight loss (average 50 kg) in their patients [50]. Whether this finding is the result of differences in experimental design, *i.e*, frequent blood sampling with deconvolution *versus* a single fasting morning specimen, is not known.

Approximate Entropy of PRL was increased in elderly men compared with subjects younger than 50 yr, but not in women. A common denominator of irregularity is attenuation of negative feedback compared with feedforward. Thus, pathophysiologies that impair feedback elevate ApEn (process randomness). Well-established conditions of high ApEn include primary failure of a target gland like the thyroid, testis, and ovary, autonomous endocrine tumors, and PCOS [55]–[61]. Additionally, excessive feedforward enforces irregular patterns. Parathyroid hormone secretion is more disorderly in hyperparathyroidism [62], as is aldosterone secretion in primary and secondary hyperaldosteronism [63]. In keeping with these observations, irregularity can be induced experimentally by muting negative feedback by testosterone, cortisol, and IGF-I, which normally maintain regularity of their upstream hormones (LH, ACTH, GH), as well as by augmenting feedforward by GnRH or GHRH on downstream hormones (LH and GH) [64]. In old age hormone secretory regularity is generally diminished, as described for GH, cortisol, and LH, but not ACTH and TSH [11]–[13], and here in elderly men for PRL, but not in women. Nonetheless, one previous study in 10 young and 10 old men found a non-significant 20% rise in PRL ApEn in elderly men [65]. Clearly, more elderly healthy subjects, with ages extending into the nineties are required to confirm ApEn changes of PRL secretion with age.

Spikiness is a measure of sharp staccato-like excursions in serial measurements, putatively reflecting acute stimuli from outside normative feedback loops [31]. In elderly men, spikiness was increased compared with younger males. In another large study by us, GH spikiness was determined jointly by gender (higher in women) and BMI (positively), accounting for 29% of inter individual variability. Spikiness also marks other endocrine physiology and pathophysiology, such as greater survival when applied to serial glucose data in patients with protracted critical illness, older age in women when applied to ACTH time series, sharper TSH excursions in hypothyroidism and thyrotropinoma compared with normal, and unstable premenstrual mood-rating dynamics in dysphoria and response to therapy in the latter group [13], [66]–[68]. Recognition of increased spikiness in older adults is important in planning investigations in this age group because greater spikiness would decrease statistical power by accentuating single-sample variability in aging individuals.

As expected, and concordant with the literature, the mesor and amplitude were larger in women than in men [69]. No gender difference was found for the acrophase of the PRL rhythm. A novel finding in this study was the dependence of the acrophase on BMI and age, both causing an advance shift, thus leading to an earlier maximum. This result is comparable with the advanced shift of cortisol in elderly subjects reported in other studies [70]–[72]. The mechanism(s) behind these shifts are not known, although changes in the sleep quality have been advocated [70]. However, no detailed studies on the effect of adiposity on hormone acrophases are available.

This study has several limitations. First, the retrospective design could have introduced a selection bias, because the volunteers participated in several independent studies as mentioned in the Introduction. However all subjects were recruited by advertisements from the local area. In addition all subjects underwent a structured medical history assessment, physical examination and routine clinical chemistry. The sampling protocol and chemical methods were identical in these studies and all studies were uneventful. Although we cannot completely rule out stress or anxiety in the volunteers, we did not detect this in the individual serum hormone patterns. Second, as mentioned above we did not use the more sensitive and specific high performance liquid chromatography-tandem mass spectrometry, but a reasonably sensitive, but necessarily less accurate estradiol RIA. Third, we have not included healthy volunteers aged over eighty years. Future inclusion of this cohort into the analysis may strengthen conclusions.

In summary, in healthy adults, selective combinations of gender, age, and BMI specify distinct PRL dynamics, thus requiring balanced representation of these variables in comparative PRL studies.
