# Correction: Prolactin Secretion in Healthy Adults Is Determined by Gender, Age and Body Mass Index

**DOI:** 10.1371/journal.pone.0110203

**Published:** 2014-09-30

**Authors:** The PLOS ONE Staff

There are errors in Table 3. Please see the corrected Table 3 here.

**Table 3:** Prolactin secretion characteristics in premenopausal and postmenopausal women, and men.

	Men	Premenopausal women	Postmenopausal women	ANOVA P-value	Men vs postmp women. P-value	Premp vs postmp women P-value
BMI	25.2±3.50	28.5±6.39	25.2±3.67	0.12		
Pulse frequency (nr/24h)	19.5±4.0	18.9±4.5	21.8±3.1	0.13		
Slow half-life (min)	33.2±7.8	34.3±8.3	32.4±9.1	0.80		
Mode day (min)	10.9±5.2	10.4±5.1	8.1±5.6	0.29		
Mode night (min)	11.2±4.8	12.8±6.4	10.4±5.4	0.92		
Basal secretion (µg/L.24h)	106±57	163±138	128±82	0.73		
Pulsatile secretion (µg/L.24h)	101±46	203±147	113±34	<0.001	0.30	<0.001
Total secretion (µg/L.24h)	207±82	366±188	241±82	<0.001	0.31	0.02
Mean pulse mass (µg/L)	5.3±2.53	10.6±1.17	5.17±1.77	<0.001	0.74	0.001
Lambda (pulse frequency)	18.1±3.6	17.6±3.9	19.5±3.5	0.25		
Gamma (regularity, unitless)	1.943±0.352	2.017±0.433	2.138±0.427	0.06		
ApEn (unitless)	1.032±0.314	0.930±0.349	1.023±0.337	0.32		
Spikiness (unitless)	0.391±0.130	0.363±0.143	0.381±0.127	0.56		
Mean 24 h PRL (µg/L)	4.35±1.46	7.72±3.16	5.10±2.09	<0.001	0.27	0.001
Minimum PRL(µg/L)	2.13±1.03	3.49±2.16	2.52±1.09	0.005	0.46	0.08
Maximum PRL (µg/L)	10.7±4.04	20.3±7.07	11.3±4.56	<0.001	0.75	<0.001
Fasting PRL (µg/L)	4.18±1.51	7.01±5.17	3.88±1.42	0.001	0.48	0.002

Data are shown as mean ± standard deviation. Data were logarithmically transformed before the ANOVA.

## References

[1] Roelfsema F, Pijl H, Keenan DM, Veldhuis JD (2012) Prolactin Secretion in Healthy Adults Is Determined by Gender, Age and Body Mass Index. PLoS ONE 7(2): e31305 doi:10.1371/journal.pone.0031305 22363612 10.1371/journal.pone.0031305 PMC3281966

